# Coffee Silverskin Extract: Nutritional Value, Safety and Effect on Key Biological Functions

**DOI:** 10.3390/nu11112693

**Published:** 2019-11-07

**Authors:** Amaia Iriondo-DeHond, Maria Belen Rios, Teresa Herrera, Antonio Rodriguez-Bertos, Fernando Nuñez, Manuel Ignacio San Andres, Sebastian Sanchez-Fortun, Maria Dolores del Castillo

**Affiliations:** 1 Instituto de Investigación en Ciencias de la Alimentación (CIAL) (CSIC-UAM), Calle Nicolas Cabrera 9, 28049 Madrid, Spain; amaia.iriondo@csic.es (A.I.-D.); belen.rios@csic.es (M.B.R.); teresaherrera60@hotmail.com (T.H.); 2 Department of Internal Medicine and Animal Surgery, School of Veterinary Sciences, Health Surveillance Center (VISAVET), Complutense University, Puerta de Hierro Ave, 28040 Madrid, Spain; arbertos@visavet.ucm.es; 3 Facultad de Veterinaria, Universidad Complutense de Madrid, Av. Puerta de Hierro, s/n, 28040 Madrid, Spain; misanand@ucm.es (M.I.S.A.); fortun@ucm.es (S.S.-F.); 4 Centro de Biología Molecular Severo Ochoa (CBMSO, CSIC-UAM), Calle Nicolás Cabrera, 1, 28049 Madrid, Spain; fnunez@cbm.csic.es

**Keywords:** antioxidant, coffee silverskin, food safety, novel food, nutrition, short-chain fatty acids

## Abstract

This study aimed to complete the scientific basis for the validation of a coffee silverskin extract (CSE) as a novel food ingredient according to European legislation. Nutritional value, safety, effects on biochemical biomarkers and excretion of short chain fatty acids (SCFAs) in vivo of CSE were assessed. Proteins, amino acids, fat, fatty acids, fiber, simple sugars and micronutrients were analyzed. For the first time, toxicological and physiological effects were evaluated in vivo by a repeated-dose study in healthy Wistar rats. Hormone secretion, antioxidant (enzymatic and no-enzymatic) and anti-inflammatory biomarkers, and dietary fiber fermentability of CSE (analysis of SCFAs in feces) were studied in biological samples. This unique research confirms the feasibility of CSE as a human dietary supplement with several nutrition claims: “source of proteins (16%), potassium, magnesium, calcium and vitamin C, low in fat (0.44%) and high in fiber (22%)”. This is the first report demonstrating that its oral administration (1 g/kg) for 28 days is innocuous. Hormone secretion, antioxidant or anti-inflammatory biomarkers were not affected in heathy animals. Total SCFAs derived from CSE fiber fermentation were significantly higher (*p* < 0.05) in male treated rats compared to male control rats. All the new information pinpoints CSE as a natural, sustainable and safe food ingredient containing fermentable fiber able to produce SCFAs with beneficial effects on gut microbiota.

## 1. Introduction

According to the European Food Safety Authority (EFSA), a ‘novel food’ is defined as “any food that was not used for human consumption to a significant degree within the Union before 15 May 1997 irrespective of the dates of accession of Member States to the Union” [1]. In this sense, coffee silverskin (CS), a thin tegument of the outer layer of the coffee beans released during coffee roasting [2], may fall under the category of “food consisting of, isolated from or produced from plants or their parts”. The application for authorizing the placement of a novel food on the market within the European Union and updating the Union list should include the name and description of the novel food, a description of the production process and the detailed composition of the novel food [1]. Based on its nutritional and chemical composition, CS could potentially be used as a food ingredient for human consumption within the concept of a healthy diet [3].

The major component in CS is dietary fiber (up to 55%), which includes insoluble (≈45%) and soluble (≈10%) fiber [4,5]. Dietary fiber is one of the main nutritional factors contributing to human well-being [6]. The EFSA defines dietary fiber as non-digestible carbohydrates, including non-starch polysaccharides, resistant starch and oligosaccharides and lignin [7]. According to the e-Library of Evidence for Nutrition Actions (eLENA) created by the World Health Organization (WHO), dietary fiber has important beneficial physiological effects that may contribute to reducing the risk of non-communicable chronic diseases [8]. In the large intestine, dietary fiber is fermented by the microbiota, leading to the generation of short-chain fatty acids (SCFAs) that also contribute to human well-being [9]. The high dietary fiber content of CS might benefit the intestine and gut microbiota [3,4,5]. Previous studies have reported the prebiotic properties of this by-product, but the effect of CS intake on the excretion of SCFAs has not been previously described [10].

The second major component present in CS is protein (≈19%), followed by carbohydrates (≈6%) and fat (≈2%) [3]. CS is also a source of polyphenols, particularly chlorogenic acid (CGA) (588.9 mg/100 g) of which 5-O-, 3-O- and 4-O-caffeoylquinic acids are the most relevant [11]. This by-product also contains caffeine (1%) and melanoidins (5%)—the latter formed during the roasting process [10,12].

According to European legislation, a ‘health claim’ means any claim that states, suggests or implies that a relationship exists between a food category, a food or one of its constituents and health [13]. However, the evaluation of the biological effects of foods and their impact on health is a limitation in nutrition studies. Nutritional biomarkers are used to objectively measure the nutritional status with respect to the intake or metabolism of dietary constituents in different biological samples [14]. These biomarkers also evaluate the bioavailability of a nutrient, which is determined by the biochemical analysis of its metabolites [14]. The study of the metabolism of the bioactive compounds, caffeine and chlorogenic acid, present in an aqueous extract of CS (CSE) has been previously studied [15], but to the best of our knowledge, the analysis of nutritional biomarkers after the intake of CSE has not been addressed yet.

All novel foods must be scientifically proven to be safe to public health, and scientific evidence demonstrating that the novel food does not pose a safety risk to human health should be included in the application for authorization [1]. Data regarding the safety of the novel food such as toxicological studies, including repeated-dose toxicity, are required. The main objective of repeated-dose toxicity studies is to identify any adverse effects following prolonged exposure via an appropriate oral route [16].

Therefore, this research aimed to complete the scientific basis for the validation of a coffee silverskin extract (CSE) as a novel food ingredient according to European legislation. To achieve this goal, a repeated-dose study was carried out in healthy Wistar rats, and hormone secretion, antioxidant and anti-inflammatory biomarkers and the fiber effect of CSE were analyzed in biological samples. Considering previous studies regarding the effect of CSE on carbohydrate metabolism [17], insulin secretion was measured after the repeated intake of this extract. As CSE contains caffeine and caffeine consumption leads to a decreased secretion of melatonin [18,19], serotonin and melatonin levels were analyzed in blood samples of the rats. Since CSE is known for its antioxidant capacity and oxidative stress and inflammation are closely related, antioxidant and anti-inflammatory biomarkers were also measured in the rat’s serum samples.

## 2. Materials and Methods

### 2.1. Coffee Silverskin Extract (CSE)

Coffee silverskin from Arabica species was provided by Fortaleza S.A. (Spain). CSE was produced as described in patent WO 2013/004873 [20]. Briefly, 50 g of coffee silverskin were added per H_2_O liter. This mixture was stirred for 10 min at 100 °C, filtered, and the filtrate was freeze-dried. The extract (yield = 14% *w*/*w* of the initial sample) was stored at −20 °C until analysis.

### 2.2. Nutritional Characterization of CSE

#### 2.2.1. Protein Content and Amino acid Composition

Protein content was determined by Kjeldahl mineralization followed by a colorimetric analysis of nitrogen for quantification (AOAC-32.1.22, 920.87). The results are expressed as % dry matter.

An amino acid analysis was performed using the AOAC-994.12 method, which is based on acid hydrolysis of the sample followed by HPLC with post column derivatization using ninhydrin. An analysis in triplicate was performed and the results are expressed as mg/g.

These analyses were performed according to that previously described [21].

#### 2.2.2. Fat and Fatty Acid Profile

Total fat content was quantified by Soxhlet extraction with petroleum ether following the procedure described by the AOAC Official Method 945.16. The results are expressed as % dry matter.

Fatty acid profile was obtained by gas chromatography (Agilent 7820A GC System equipped with Flame Ionization Detector) analyses [22], calculated according to the ISO 12966-2:2017.

These analyses were performed according to that previously described [23].

#### 2.2.3. Soluble Simple Sugars

Determination of sugars (mannose, glucose, xylose, fructose and sucrose) was carried out by ion exchange liquid chromatography (LC-IC). The LC system used in this study was a Metrohm Advanced Compact ion chromatographic instrument (Metrohm, Switzerland) with a Pulsed Amperometric Detector (PAD) (Bioscan module, 817 IC. Metrohm), a pump (IC Pump 812), a coupled degasser (IC-837) and a Metrosep Carb 2 column (250 × 4 mm) packed with 5-μm spherical polymer beads. A mobile phase consisting of 100 mM NaOH and 10 mM NaOAc was applied at a flow rate of 0.5 mL/min, and 20 μL of sample were injected in the LC-IC system. Data were analyzed using Metrodata IC Net 2.3 software (Metrohm, Switzerland). This quantification was performed by the Analysis Service Unit facilities of the Institute of Food Science, Technology and Nutrition (ICTAN, CSIC, Madrid).

#### 2.2.4. Dietary Fiber

Insoluble (IDF), soluble (SDF) and total (TDF) dietary fiber were determined using an enzymatic-gravimetric assay based on the AOAC-991.43 and AACC-32.07.01 methods. The results are expressed as %.

#### 2.2.5. Ions and Ascorbic Acid

Determination of ions (sodium, potassium, magnesium and calcium) was carried out by ion exchange liquid chromatography (LC-IC) Metrohm Advanced Compact ion chromatographic instrument (867 IC. Metrohm, Switzerland) with a 819 Advanced IC Detector, a pump (IC Pump 818), a coupled degasser (IC-837) and a Metrosep C6 column (250 × 4 mm) packed with 5-μm spherical polymer beads (Metrohm AG, Switzerland). A mobile phase consisting of 3 mM metasulfonic acid was applied at 1 mL/min and 20 μL of sample were injected in the LC-IC system. Determination of ascorbic acid was performed using the same equipment and a Metrosep Organic Acids column (250 × 4 mm, 5-μm particle size) (Metrohm AG, Switzerland). The mobile phase was 50 mM lithium chloride at a flow rate of 0.5 mL/min. Data were analyzed using Metrodata IC Net 2.3 software (Metrohm, Switzerland). This quantification was performed by the Analysis Service Unit facilities of the Institute of Food Science, Technology and Nutrition (ICTAN, CSIC, Madrid).

### 2.3. Repeated-Dose Study

Healthy young adult male (*n* = 15) and female rats (*n* = 15) (3 weeks old) (*Rattus norvegicus albinus*), weighing 67.2 ± 4.7 g at the start of the experiment, were obtained from Charles River (Sant Cugat del Vallés, Spain). The animals were acclimated for seven days prior to the beginning of the experiment. The rats were housed in cages with a maximum capacity of five animals per cage under a controlled temperature (22 ± 1 °C) in a 12-h light/dark cycle with free access to standard food (A04 Safe Diets, Augy, France) and water *ad libitum*. As it was not possible to replace the use of animals, all our effort was directed at the minimization of the number of animals and any possible discomfort caused to them. The experimental protocols used were previously approved by the Ethics Committee on Animal Use (CEEA). The present study was approved by the Institutional Animal Ethics Committee (Reg. No. PROEX 011/17) of Community of Madrid, Spain.

The repeated dose toxicity study was carried out in accordance with OECD Test Guidelines 407 (Repeated Dose 28-day Oral Toxicity Study in Rodents) [24]. The highest dose level was chosen to induce toxic signs. Thus, the dose level of powdered CSE in aqueous solution was 1 g/kg body weight (b.w.) per day based on data from previous studies [19]. Rats were randomly allocated to two groups, the control or the group treated with CSE. The control group consisted of 7 males and 7 females, and the treated group had 8 males and 8 females. Animals received sample (CSE 1 g/kg b.w.) or the vehicle (water) once daily by oral gavage for a total of 28 days. The dose volume was set at 1 mL/100 g b.w. Throughout the experiment, animals of both groups were observed daily before initiating administration and once a week during the administration period, and detailed clinical signs were recorded. Body weight, food and water intake, behavior and signs of toxicity were recorded daily from the start to the 28th day of the experiment.

#### 2.3.1. Safety

##### Necropsy, Macroscopic Analysis and Organ Weight

At the end of the treatment with CSE (1 g/kg b.w.), male and female rats were killed 1 day after the 28th administration. At the time of necropsy, animals were previously anesthetized with isoflurane (Forane^®^, 4% induction dose using oxygen as a carrier gas and 2% for maintenance during the process) and then, total blood was collected by cardiac puncture. Rats were then sacrificed by exposure to excess carbon dioxide in a gas chamber. After euthanasia, macroscopic assessment of the external body surface, cavities (thoracic, abdominal and cranial) and organs (change in position, shape, size, color and consistency) was carried out. Absolute and relative weight of the brain, lungs, liver, heart, spleen, thymus, kidneys, adrenal glands and sex organs was also determined. Relative organ weight was calculated using the following formula:

Relative organ weight (g %) = (weight of organ/weight of rat) × 100.

##### Histopathological Examination

Tissue samples from the brain, lungs, liver, heart, spleen, thymus, kidneys, adrenal glands and sex organs were routinely processed for histology and fixed in buffered formalin 10% (Panreac©, Barcelona, Spain, stabilized with methanol at pH 7) for 24 h at room temperature. Samples were then embedded in synthetic paraffin (Casa Alvarez, Madrid, Spain) with a melting point of 56 °C, using an automatic tissue processor (ASP300, Leica^®^, Wetzlar, Germany) with a program of automatic transmissions alcohols increased histological grading. Blocks were performed in a block forming unit (console Leica© EG1140H and cold plate Leica© EG1130), and 4 micron thick sections were obtained from rotation microtome Leica© brand, model RM 2155. Sections were deparaffinized in xylene and hydrated in alcohol and water. Conventional staining method, hematoxylin and eosin, was used by means of Leica© auto stainer SP4040. Then, dehydrated first ascending alcohol series and xylene were used, and finally, mounted with DPX (Nustain©, Nottingham, UK). The slides were then observed under a light microscope.

##### Hematological and Biochemical Parameters

Blood samples were collected by cardiac puncture of the anesthetized animals and stored in tubes with anticoagulant for determination of hematological parameters, and in tubes without anticoagulant to obtain serum for determination of biochemical parameters. Laser flow cytometry technology (ADVIA 120 Hematology System, Siemens Healthineers, Getafe, Spain) was used to examine the following parameters: red blood cells (RBCs), hemoglobin concentration, hematocrit (HCT) value, mean corpuscular volume (MCV), mean corpuscular hemoglobin (MCH), mean corpuscular hemoglobin concentration (MCHC), red cell distribution width (RDW), white blood cells (WBCs), platelet count, mean platelet volume (MPV) and plateletcrit. Differential leukocyte count was determined by blood smear.

Measurements of plasma concentration, glucose, urea, creatinine, alanine-transferase (ALT), γ-glutamyl-transferase (GGT) and total cholesterol were performed by reflectance photometry (Reflotron^®^, Roche Diagnostics, Germany). The serum concentration of total proteins, albumin and bile acids was determined by liquid spectrophotometry (Biuret Test, bromocresol green technique and 3-α-hydroxysteroid dehydrogenase enzymatic technique, respectively), while the ionogram (sodium, potassium) was obtained by means of a blood gas analyzer (ABL90 Flex, Radiometer Medical ApS, Brønshøj, Denmark).

#### 2.3.2. Effect on key Biological Functions

##### Hormone Levels

Insulin levels were determined by a sandwich enzyme immunoassay kit (RayBio^®^ Rat Insulin ELISA, Norcross, USA) according to the manufacturer’s instructions. The kit was characterized by an analytical sensitivity of 5 μIU/mL and high analytical specificity (low-cross reactivity).

Serotonin levels were determined by a competitive enzyme immunoassay kit (General Serotonin Elisa Kit SAB Laboratories) according to the manufacturer’s instructions. The kit was characterized by a detection range of 1.56–100 ng/mL and no significant cross reactivity or interference was observed.

Melatonin levels were determined by a competitive enzyme immunoassay kit (Rat MT (Melatonin), Elabscience, Wuhan, China) according to the manufacturer’s instructions. The kit was characterized by an analytical sensitivity of 9.38 pg/mL, a detection range of 15.63–1000 pg/mL and no significant cross reactivity or interference was observed.

##### Oxidative Stress Biomarkers

**Non-enzymatic antioxidant capacity**

The trapping capacity of cationic free radicals in serum samples was evaluated using the method of radical ABTS^+^ bleaching described by Re et al. (1999) [25] and modified by Oki et al. (2006) [26] for its use in microplate. Aqueous solutions of Trolox (0.15–2.0 mM) were used for calibration. Absorbance was measured in microplate using a UV–Visible Spectrophotometer (BioTek Instruments, Winooski, VT, USA). All measurements were performed in triplicate, and results were expressed as eq. Trolox (mM).

**Enzymatic oxidative stress biomarkers**

Glutathione peroxidase (GPx) activity was measured in serum samples by the Glutathione Peroxidase Activity Assay Kit (MBL International, Woburn, USA) following the manufacturer’s instructions. The assay had a detection sensitivity of ≈0.5 mU/mL of GPx in samples.

Glutathione reductase (GR) activity was measured in serum samples by the Glutathione Reductase Colorimetric Activity Assay Kit (MBL International, Woburn, USA) following the manufacturer’s instructions. The assay could detect 0.1–40 mU/mL GR in samples.

Superoxide dismutase (SOD) activity was determined by SUPEROXIDE DISMUTASE Colorimetric Activity Kit (Arbor Assays, Ann Arbor, USA) in serum samples following the manufacturer’s instructions. The sensitivity of the kit was 0.044 U/mL.

Catalase (CAT) activity was determined by CATALASE Colorimetric Activity Kit (Arbor Assays, Ann Arbor, USA) in serum samples following the manufacturer’s instructions. The sensitivity of the kit was 0.052 U/mL and the limit of detection was 0.062 U/mL.

##### Inflammation Biomarkers

C-reactive protein (CRP) levels were determined by a sandwich enzyme immunoassay kit (Rat CRP Wuhan, China) according to the manufacturer’s instructions. The kit was characterized by an analytical sensitivity of 0.19 ng/mL and high analytical specificity (low-cross reactivity).

##### Dietary Fiber Effect

Feces produced during a 24-h period were collected from each cage of the treated and control group on the last day of the study. To evaluate the fiber effect of CSE, feces were counted and weighed. In addition, pH was measured, since the level of fermentable carbohydrates and antioxidants in the diet are important factors affecting pH in feces. The antioxidant capacity of feces was determined by the ABTS^+^ method (Section “Non-enzymatic antioxidant capacity”).

Short chain fatty acids (SCFAs) were also determined in the feces from both groups. Five grams (wet weight) of rat feces were diluted in 50 mL of phosphoric acid 0.5%, homogenized with an ultra-turrax (IKA^®^-Werke GmbH & Co. KG, Germany) for one minute, frozen with liquid nitrogen and after thawing, centrifuged at 4000 rpm for 20 min, and the supernatant was filtered through a membrane filter (pore size, 0.45 µm). Each milliliter of water supernatant was extracted with 1 mL of butanol and, prior to analysis, 4-methyl valeric acid was added as an internal standard (IS). The IS was used to correct for injection variability between samples and minor changes in the instrument response. SCFAs were identified and quantified by using gas chromatography (Agilent 7890A) coupled to mass spectrometry (Agilent 5975C) (Agilent Technologies, Santa Clara, CA, USA) with a DB-WAXtr column (60 m × 0.325 mm × 0.25 µm) (Agilent Technologies) [27]. The injector, ion source, quadrupole and the GC/MS interface temperature were 250 °C, 230 °C, 150 °C and 280 °C, respectively. The flow rate of helium carrier gas was kept at 1.5 mL/min. The initial column temperature was 50 °C and held 2 min, ramped to 150 °C at the rate of 15 °C/min, to 200 °C at 5 °C/min and then finally increased to 240 °C at the rate of 15 °C/min and kept at this temperature for 20 min. Ionization was carried out in the electron impact (EI) mode at 70 eV. The analytes were quantified in the selected ion monitoring (SIM) mode using the target ion and confirmed by confirmative ions. The target ion (*m*/*z*) of the IS was 57 and of acetic, propionic, isobutyric, butyric, isovaleric, valeric, caproic and heptanoic acids are 43, 74, 43, 60, 60, 60, 60 and 60, respectively. Data were analyzed using MSD Chemstation E.02.00.493 program. The contents of SCFAs were calculated with standard methods.

### 2.4. Statistical Analysis

Data were expressed as the mean ± standard deviation. One-way analysis of variance (ANOVA) was performed and statistical comparisons of the different treatments were performed using Tukey’s test. Values of *p* < 0.05 were considered statistically significant. All statistical analyses were performed using SPSS Statistics 24 (IBM, Armonk, NY, USA).

## 3. Results

### 3.1. Nutritional Characterization

The protein content and free and total amino acids present in CSE are shown in Table 1. Protein content was 16.17% (*w*/*w*). Considering, free and total amino acids, 37% and 34% of the total corresponded to essential amino acids, respectively. Serine, asparagine and arginine presented the highest values of free amino acids, while asparagine together with glutamic acid and glycine showed the highest values for total amino acids. Free tryptophan levels found in CSE were 0.25 mg/g.

Fat content and the fatty acid profile of CSE is shown in Table S1. Fat content was 0.44 %. In this study, C:16 was the main fatty acid found (26%), followed by C18:2n6c (21%), C22:0 (19%), and C20:0 (12 %). CSE mainly presents saturated fatty acids (SFA) (67%), followed by polyunsaturated (PUFA) (26 %) and monounsaturated (MUFA) (7%) fatty acids (Table S1).

The soluble simple carbohydrates detected in CSE were fructose (3.21 g/100 g), sucrose (1.97 g/100 g), glucose (1.29 g/100 g), mannose (0.06 g/100 g) and xylose (0.05 g/100 g).

TDF detected in CSE was 21.81 ± 0.77%. Dietary fiber present in CSE is mainly composed of SDF (13.36 ± 20.3%), representing 61% of TDF in CSE. IDF values were 8.18 ± 0.17%.

As for micronutrients, potassium (5600 mg/100 g), magnesium (530 mg/100 g), sodium (460 mg/100 g) and calcium (350 mg/100 g) were also detected in this extract. In addition, the results obtained from the analysis of organic acids show values of 110 mg of ascorbic acid per 100 g of CSE.

### 3.2. Repeated-Dose Study

#### 3.2.1. Safety

No adverse clinical signs or mortality were observed in animals treated with CSE (1 g/kg b.w.) for 28 days.

##### Food and Water Intake

According to the results shown in Table 2, animals treated orally with CSE (1 g/kg b.w.) did not show significant changes in food intake compared to the control group (*p* > 0.05). After 2 weeks, male rats presented a significantly higher food intake (*p* < 0.05) than female rats. Considering the fiber content present in rat feed (3.9%) and in CSE (22%), male rats treated with CSE had a significantly increased intake of total dietary fiber (*p* < 0.05). However, this increased fiber consumption was not observed in female animals (*p* > 0.05). No significant differences (*p* > 0.05) in water intake were observed during the first 3 weeks of the study. However, during the 4th week of treatment, water intake of female rats treated with CSE was significantly higher (*p* < 0.05) than that observed for rats from the control group (Table 2).

##### Body and Organ Weight

**Figure 1:**
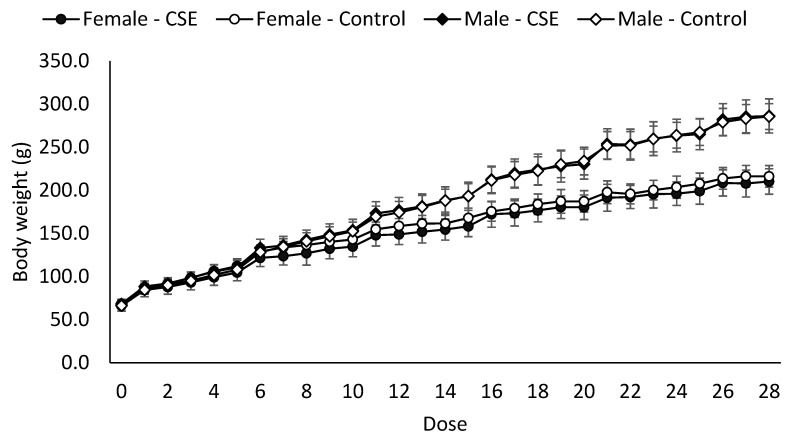
Body weight (g) of controls and rats treated with coffee silverskin extracts (CSE).

Figure 1 shows changes in absolute body weight of control and rats treated with CSE. At a dose of 1 g/kg b.w. of CSE, no significant changes were observed (*p* > 0.05) in treated rats compared to the control group up to the 28th day of the repeated dose toxicity study. After 2 weeks, treated and control male rats significantly increased (*p* < 0.05) their body weight compared to treated and control female rats. These results are in accordance with those observed in the food intake analysis (Table 2).

The mean and absolute organ weights of control male and female rats and rats treated with CSE (1 g/kg b.w.) are shown in Table 3. No significant changes were observed in any organ weight (*p* > 0.05) in animals treated with CSE compared to the control group. In addition, post-mortem exams were performed in all of the animals to macroscopically observe any disturbances in vital organs (liver, heart, lungs, kidneys, thymus, adrenal glands, sex organs, brain and spleen) and to collect tissue samples for histology (liver, heart and kidneys). There were no gross findings considered to be related to the administration of CSE. Hemorrhage was found in the lungs of control and treated rats, possibly derived from the sacrifice method employed.

##### Histopathology

**Figure 2:**
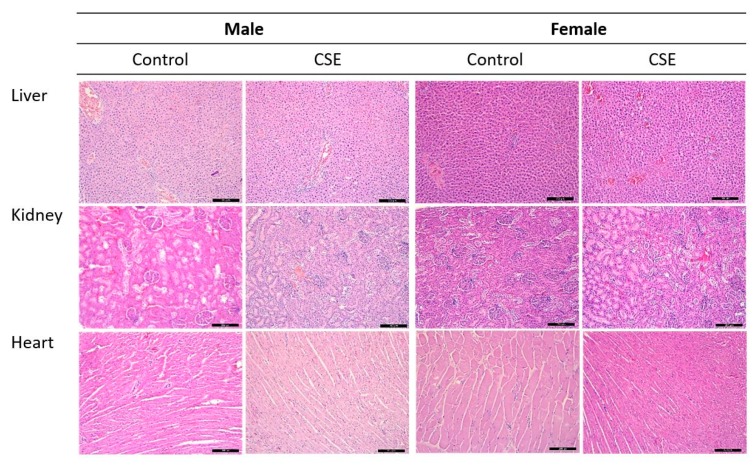
Representative microscopic findings for the liver, kidneys and heart of female and male Wistar rats treated with 1 g/kg b.w. CSE in aqueous solution and the controls for 28 days. Scale bar = 200 µm.

At the end of the study, vital organs were subjected to histopathological examination. Microscopic observation showed no remarkable pathological changes in the liver, kidneys or heart (Figure 2), or in the rest of the studied organs of rats of both sexes and treated with CSE (1 g/kg b.w.) after the repeated-dose toxicity study.

##### Hematological and Biochemical Parameters

According to the results described in Table 4, there were no statistically significant differences (*p* > 0.05) in the biochemical parameters studied in the blood samples between the rats treated with CSE (1 g/kg b.w.) compared to the control group. Similar results were found in the hematological parameters also shown in Table 4, since no significant changes were observed in erythrocyte, leukocyte and platelet parameters (*p* > 0.05) of treated rats compared to the control group.

#### 3.2.2. Effect on Key Biological Functions

##### Hormone Levels

Evaluation of insulin, serotonin and melatonin levels after the 28-day repeated dose toxicity study did not report any differences in these hormone levels (*p* > 0.05) between treated and control rats (Table 5).

##### Oxidative Stress and Inflammation Biomarkers

Table 5 shows the total antioxidant capacity (TAC) and the enzymatic oxidative stress biomarkers (glutathione peroxidase (GPx), gluthathione reductase (GR), superoxide dismutase (SOD) and catalase (CAT)) studied in serum samples of control and rats treated with CSE (1 g/kg b.w.). Daily administration of CSE (1 g/kg b.w.) did not significantly affect antioxidant biomarkers in blood samples (*p* > 0.05). Furthermore, no significant differences (*p* > 0.05) were observed in C reactive protein levels between groups, suggesting no signs of inflammation when this particular biomarker was studied.

##### Dietary Fiber Effect

With regard to the analyses carried out in feces generated during a 24-h period and collected on the last day of the study (Table 6), control male rats showed a significantly higher (*p* < 0.05) number of pellets than male rats treated with CSE. No significant differences were detected in the pellet number of female rats. Furthermore, no significant differences were detected in feces weight between groups, and treatment of male and female rats with CSE (1 g/kg b.w.) did not affect pH or total antioxidant capacity (*p* > 0.05) of rat feces collected at the end of the study (Table 6).

The results of SCFAs analyzed in feces collected on the last day of the study are shown in Table 6. The most abundant SCFAs found in feces were acetate, propionate and butyrate. Total SCFAs (60 μmoL/g and 78 μmoL/g for control and treated rats, respectively) were significantly greater (*p* < 0.05) in feces when male rats were treated with CSE. However, this effect was not observed in female rats (79 μmoL/g and 73 μmoL/g for control and treated female rats, respectively). In male rats, acetate significantly increased (*p* < 0.05) in feces after the repeated intake of CSE.

## 4. Discussion

### 4.1. Nutritional Quality

Considering the nutritional profile of CS, this by-product has a great potential for use as a novel food ingredient [10,28,29,30]. The results obtained in the present study suggest that the aqueous extract obtained from CS can be considered a ‘source of proteins’ since it contains over 12 g/100 g of proteins as stated in Regulation (EU) No 1924/2006 [13] (Table 1). Protein values of CSE are similar to those of egg (12%) and higher than the protein content in milk (3%), as reported by the United States Department of Agriculture (USDA) National Nutrient Database. According to Commission Regulation (EU) No 432/2012, the health claims for foods that are a source of proteins could be applied to CSE. These health claims are as follows: ‘protein contributes to the growth and maintenance of muscle mass’ and ‘to the normal maintenance of normal bones’ [31]. The extract did not have a significant effect (*p* > 0.05) on protein intake biochemical parameters determined in blood samples from rats, urea and creatinine, compared to control rats (Table 4). The values obtained for these biomarkers were within the physiological range [32].

To the best of our knowledge, this is the first study that shows the amino acid profile of CSE. The proportion of essential amino acids (34–37%) makes CSE a good source of indispensable amino acids (Table 1). The claimed effects related to amino acids are growth or maintenance of muscle mass, maintenance of normal muscle function, faster recovery of muscle function/strength/glycogen stores after exercise, faster recovery from muscle fatigue after exercise and skeletal muscle tissue repair [33]. However, these claimed effects are not established in terms of a cause–effect relationship and have been evaluated by the EFSA Panel with an unfavorable opinion [33]. CSE may be used as a novel ingredient in foods as a source of amino acids.

As the fat content of CS was 0.44%, this extract could also be considered a ‘low fat’ product [13]. Fat content in CSE was lower than previously described for CS, which ranged from 1.6% to 3.3% [3,34]. Fatty acid composition of CS mainly includes palmitic acid (C16:0), followed by linoleic acid (C18:2n6) and behenic acid (C:22:0), which agrees with that previously reported by other authors [35]. As expected, the repeated intake of CSE (1 g/kg b.w.) did not have an effect on fat consumption biomarkers, such as cholesterol, aminotransferases and bile acids (Table 4) in rats treated with CSE compared to the control group.

The total amount of simple carbohydrates composing CSE (6.58 g/100 g) did not rise (*p* > 0.05) the glucose levels of treated rats compared to control rats (Table 4). The amount of total simple sugars is close to the value stated by the European Commission of 5 g of sugars per 100 g for reaching the nutrition claim of ‘low sugar’ [13]. The value of simple sugars obtained in this study is similar to that obtained by Costa et al. (2018) [3]. In contrast, this value is higher than that reported for a mixture of Arabica and Robusta CS by Toschi et al. (2014), although the main simple carbohydrates detected in both studies were fructose and sucrose [35].

The amount of total dietary fiber obtained in this study (22%), as well as SDF (14%) and IDF (8%), agrees with that previously reported for CSE (28%, 24% and 4% for TDF, SDF and IDF, respectively) [11]. Different authors have reported values of approximately 60%, 50% and 8% of total, insoluble and soluble dietary fiber of CS, respectively [3,5,10,28]. Ballesteros et al. (2014) reported that insoluble dietary fiber in CS is composed of cellulose, hemicellulose and lignin [5]. This potential novel ingredient can reach the nutrition claim of ‘high in fiber’ whereby the product must contain at least 6 g of fiber per 100 g [13]. The health claims attributed to the “high in fiber” nutrition claim are ‘fiber increases fecal bulk, contributes to normal bowel function and to an acceleration of intestinal transit’ [31].

The WHO recommends a potassium intake of at least 90 mmol/day (3.5 g/day) for adults to reduce blood pressure and the risk of cardiovascular disease, stroke and coronary heart disease [36]. In this sense, CSE would be a good source of potassium (5.6 g/100 g) [37], in accordance with Costa et al. (2018) [3]. According to Commission Regulation (EU) No 432/2012, foods that are a source of potassium can be labeled under the following health claims: “potassium contributes to normal functioning of the nervous system, to normal muscle function and to normal blood pressure” [31].

On the other hand, the studied extract may also be considered a ‘source of magnesium, calcium and vitamin C’. Recommended daily allowances (RDAs) for magnesium, calcium and vitamin C are 300 mg, 800 mg and 60 mg, respectively [37]. Since values of magnesium, calcium and vitamin C present in CSE represent 15% of the recommended allowance per 100 g of product, this by-product may be considered a source of these compounds. The nutrition claim source of magnesium is related to the following health claims: “Magnesium contributes to a reduction of tiredness and fatigue, to electrolyte balance, to normal energy-yielding metabolism, to normal functioning of the nervous system, to normal muscle function, to normal protein synthesis, to normal physiological function, to the maintenance of normal bones and teeth and a role in the process of cell division” [31].

Calcium is also involved in the health claims: “Calcium contributes to normal blood clotting, to normal energy-yielding metabolism, to normal muscle function, to normal neurotransmission, to normal function of digestive enzymes, has a role in the process of cell division and specialization and is needed for the maintenance of normal bones and teeth” [31].

Finally, health claims regarding vitamin C content are: “Vitamin C contributes to normal function of the immune system, to normal collagen formation for the normal function of blood vessels, bones, cartilages, skin and teeth; contributes to normal energy-yielding metabolism, to normal functioning of the nervous system, to normal physiological function, to the protection of cells from oxidative stress, to the reduction of tiredness and fatigue, to the regeneration of the reduced form of vitamin E and increases iron absorption” [31].

The strong correlation between diet and health, together with sedentary lifestyles, an aging population and increasing healthcare costs have driven the interest of research in developing healthier food products [38]. Fiber-enriched foods have been developped in recent years to increase dietary fiber consumption to reduce the risk of chronic diseases. CS has been employed as dietary fiber to reduce caloric density and increase the dietary fiber content of breads [30]. CS has been added to bread formulations as a natural sustainable source of antioxidants, α-glucosidase inhibitors and colorants [39]. It has also been used as a coloring and as a dietary fiber source to achieve healthier, nutritious and high sensorial quality biscuits. The nutritional value and appearance of the biscuits also improved by the addition of CS [40]. CS has also been used in cakes, formulated with up to 30% of water-treated CS as a flour substitute [12,41]. In addition, anti-obesity and antioxidant beverages have been developed with CSE. Beverages made from Arabica and Robusta CSE (100 µg/mL) reduced body fat by 21% and 24%, respectively, in *Caenorhabditis elegans* as an animal model [28]. Finally, recent studies have used CS in yogurt production and showed that the bioactive compounds are still bioaccessible after the digestion process [42].

### 4.2. Safety

To the best of our knowledge, no study regarding the effects of prolonged exposure to an aqueous extract of CS has been performed. In the present study, CSE from Arabica coffee beans was evaluated in Wistar rats exposed to 1 g/kg b.w. The feeding of CSE (1 g/kg b.w.) to male and female Wistar rats for 4 weeks did not cause mortality in any of the animals. The absence of mortality is considered a positive aspect to support safe use of CSE in animals and in subsequent clinical trials with formulations containing this material.

The safety of these novel foods must be scientifically proven for human consumption. Previous studies have proven the safety CSE at different levels through genotoxicity and acute toxicity studies [19,43]. CSE did not induce either cytotoxicity or genotoxicity and protected human cells from DNA strand breaks and oxidative DNA damage effects of chemicals agents such as benzo(a)pyrene [43]. Furthermore, no lethal effects were observed in acute toxicity studies when rats were treated with 2 g/kg b.w. by oral administration [19].

With regard to food and water intake, there were no significant differences (*p* > 0.05) between treated and control groups, which indicates that CSE does not interfere with these parameters. Weight loss is considered of toxicological importance when the reduction is at least 10% less than the initial body weight [44]. As expected from the results obtained for food intake, the body weight of rats treated with CSE did not change compared to the control group during the 28 days of the study (*p* > 0.05), suggesting that CSE does not compromise nutrient absorption. Other authors have reported the absence of changes in food intake and body weight between control rats and rats treated with β-glucans extracted from barley for 28 days [45]. El Kabbaoui et al. (2017) also studied the effect of a vegetal aqueous extract on Wistar rats, and no significant changes in body weight were reported when rats were fed with 1 g/kg of *Cistus ladaniferus* L. extract [46].

Significant changes in absolute and relative organ weight of rats can be considered important evidence of toxicity [47]. The organs in this study were selected according to the Society of Toxicologic Pathology (STP) recommendations. With regard to the macroscopic morphological analysis, there was no change in the shape or weight of the studied organs (thymus, lungs, liver, kidneys, adrenal glands, sex organs, brain, heart and spleen) in rats treated with CSE (1 g/kg b.w.). Furthermore, the histopathology examination showed no histopathological lesions in the liver, kidneys or heart after treatment with CSE. The assessment of histopathological alterations in organs is considered a basic test in the safety assessment of tested materials [48]. These results reinforce the findings of the absence of toxicity after treatment with 1 g/kg b.w. of this compound for 28 days. Other authors have also reported the absence of morphological or histopathological alterations when Wistar rats were treated with different plant extracts following the same procedure as that described in this study [46,49,50].

The analysis of blood parameters in animal models has a high predictive value for alterations of the hematological system in human toxicity [51]. No significant differences (*p* > 0.05) were observed in biochemical parameters when the diet of rats was supplemented with CSE at 1 g/kg b.w. Glucose, cholesterol, proteins, potassium, sodium, albumin and bile acid levels in treated and control rats were in the same range of that previously described for Wistar rats [32,52,53]. Kidney and liver functionality were evaluated by the measurement of urea and creatinine, and ALT and GGT, respectively. No significant changes (*p* > 0.05) were observed in these parameters when animals were treated with CSE 1 g/kg b.w. and the obtained values were similar to those in the reference databases [32,52,53]. No signs of acute of prolonged hepatotoxicity were observed since liver enzyme levels neither increased nor decreased [54]. These results are in accordance with the absence of toxicity observed in the histopathological analysis of kidney and liver tissue of rats treated with CSE 1 g/kg b.w. for 28 days. On the other hand, damage or destruction of blood cells negatively affects the normal functioning of the body in both humans and animals [48]. The analysis of erythrocytes, leukocytes and platelets showed the absence of alterations in these parameters after treatment with CSE as well.

### 4.3. Effect on Key Biological Functions

With regard to hormone secretion, no significant differences (*p* > 0.05) were observed in insulin serum levels of healthy rats treated with CSE 1 g/kg for 28 days (Table 5). However, CSE has shown the ability to modulate insulin secretion in vitro in pancreatic INS-1E cells [55]. Doses of CSE of 1–10 μg/mL stimulated insulin secretion and reinforced antioxidant defense in pancreatic beta cells stressed with streptozotocin [15]. Previous studies have also shown the anti-diabetic properties of CSE [15,55]. Daily administration of CSE before the induction of diabetes with streptozotocin–nicotinamide (type 2 diabetes model) significantly reduced (*p* < 0.05) pancreatic oxidative stress, protecting rats from developing diabetes [15].

The essential amino acid tryptophan, which is present in CSE (Table 1), is the precursor of several important products, including serotonin or melatonin [56]. Serotonin and melatonin are both hormones that regulate various biological functions, such as sleep, appetite and mood. The melatonin-serotonin pathway affects appetite and digestive processes by endocrine as well as paracrine effects in both the brain and the gastrointestinal tract [57]. The CSE used in this study has 3.4 mg/g dry matter of melatonin [58]. However, no significant changes (*p* > 0.05) were observed in melatonin levels or in its precursor, serotonin, in the serum of rats treated with CSE compared to the control group. CSE may be used as a source of tryptophan, which will act as a neurometabolite.

The caffeine content in CSE is 24 mg/g [19], and it has been reported that caffeine also impacts circadian rhythms [59]. In humans, acute caffeine intake has been shown to delay the onset of melatonin secretion and decrease nighttime melatonin levels [60]. The results obtained in this study indicate that the physiological functions determined by these hormones were not affected by the repeated intake of CSE at 1 g/kg b.w.

Although CSE is known to have a high antioxidant capacity [10], non-enzymatic and enzymatic oxidative stress biomarkers (GPx, GR, SOD and CAT) analyzed in serum samples were not altered after the administration of CSE 1 g/kg for 28 days (Table 5). In contrast, previous studies have shown the antioxidant properties of CSE in vivo in pancreatic tissue samples of diabetic rats [61]. Further studies should be carried out to investigate the antioxidant properties of CSE in certain organs, since polyphenols are metabolized in tissues and these metabolites can also have antioxidant properties [62]. In addition, no significant differences (*p* > 0.05) were observed in inflammation biomarkers, C reactive protein, in treated rats compared to the control group. Recent studies have reported that phenolic compounds composing CSE possess anti-inflammatory properties in vitro. CSE reduced the expression of inducible nitric oxide synthase (iNOS) and cyclooxygenase-2 (COX-2) and decreased the secretion of pro-inflammatory factors in LPS-stimulated RAW2643.7 macrophages [63]. Further research regarding the analysis of several pro-inflammatory biomarkers should be carried out in vivo.

Considering the potential fiber effect of CSE, no significant changes (*p* > 0.05) in feces number or weight were observed in female rats between groups after 28 days of CSE ingestion (Table 6). However, male rats treated with CSE showed a lower number of feces compared to the control group. Previous studies have shown that the high molecular weight of CSE, rich in melanoidins, accelerated intestinal transit in treated animals after 28 days of exposure [64].

CS contains higher amount of soluble dietary fiber compared to other materials, and therefore, can be fermented, and it possesses a large water retention capacity, promotes the growth of bifidobacteria, and decreases the absorption of fat and sugars [5]. Results regarding the analysis of SCFAs showed that sex seems to influence the SCFAs profile of rats’ feces. Total SCFAs were significantly higher (*p* < 0.05) in female control rats compared to male control rats. SCFAs values and molar ratios of acetate:propionate:butyrate (which are also an indicator of dietary changes) of male rats obtained in this study are similar to those previously described [65]. Shastri et al. (2015) have previously reported the influence of sex in gut fermentation. These authors illustrated the need of considering sex in research studies that investigate health impacts from the intake of functional foods or ingredients that contribute to the improvement of gut health [66]. Male rats treated with CSE showed a significantly higher (*p* < 0.05) content of total SCFAs and acetate than male control rats. This is in agreement with the increased fiber consumption in male rats (Table 2). The higher levels of SCFAs had no significant effects (*p* > 0.05) on the pH values obtained in the feces of rats treated with CSE (Table 6). Health-promoting properties associated to acetate are increased colonic blood flow and enhanced ileal motility [67]. Considering the results obtained from the dietary fiber effect and the health claim made on foods high in fiber, it could be said that the dietary fiber present in CSE may contribute to normal bowel function.

With regard to butyrate, there is increasing evidence that this SCFA per se may be beneficial for human health [68]. Lower values of butyrate (*p* < 0.05) were observed in animals treated with CSE. The majority of SCFAs are rapidly absorbed by the colonocytes in the cecum and large intestine, and only approximately 5% are secreted in the feces. The major part of butyrate is used as fuel for colonocytes and it plays an important role in maintaining colonic health in humans [69,70]. Therefore, the butyrate produced by fermentation of the fiber present in CSE may have been absorbed by intestinal cells where it exerts potential health benefits. Fiber from another coffee by-product, spent coffee grounds (SCGs), has shown anti-inflammatory properties after in vitro fermentation by human gut microflora. SCFAs produced by colonic fermentation of SCGs exhibited great anti-inflammatory properties by suppressing NO production, and they inhibited inflammatory cytokines [71]. Considering the results obtained in this study, CSE might be considered a sustainable novel food ingredient with health-promoting properties and beneficial effects on gut microbiota.

## 5. Conclusions

The nutritional profile of CSE makes it a good candidate for use as a novel food ingredient. This novel ingredient is a source of proteins, low in fat and high in fiber. It can also be considered a source of potassium, magnesium, calcium and vitamin C. Oral administration of CSE at a dose of 1 g/kg for a period of 28 days is safe in rats. Effects on key biological functions of CSE were studied in vivo in healthy rats, and the supplementation of the diet with CSE had no negative effects on hormone secretion, antioxidant or anti-inflammatory biomarkers. The dietary fiber effect of CSE was observed since total SCFAs and acetate derived from CSE fiber fermentation were significantly higher (*p* < 0.05) in treated male rats compared to male control rats. The same trend was observed in female rats for acetic acid. Overall, coffee silverskin extract can be considered a natural, sustainable and safe food ingredient with potential effects for gastrointestinal health due to the metabolites (SCFAs) derived from the fermentation of its dietary fiber. Novel data reported in the present manuscript regarding the nutritional information, toxicology and effects on key biological functions in healthy animals, complete the scientific basis mandatory for its authorization as a novel food ingredient. In addition, they confirm its feasibility as a unique sustainable dietary supplement for human consumption. Clinical trials should be performed in the future to confirm the effects of CSE on human key biological functions and health claims for products containing the novel ingredient.

## Figures and Tables

**Table 1:** Total protein (%) and free and total amino acid content (mg/g) of coffee silverskin extract (CSE).

	CSE	
Total Protein (%)	16.17 ± 0.06	
Amino acids (mg/g)	Free	Total
Alanine (Ala)	0.66 ± 0.01	2.83 ± 0.13
Arginine (Arg)	0.79 ± 0.01	1.14 ± 0.06
Asparagine (Asp)	0.92 ± 0.02	6.41 ± 0.34
Cysteine (Cys)	0.18 ± 0.02	0.35 ± 0.01
γ-Amino butyric acid (GABA)	0.31 ± 0.00	N.D.
Glutamic acid (Glu)	0.77 ± 0.00	5.61 ± 0.31
Glycine (Gly)	0.23 ± 0.00	3.11 ± 0.14
Histidine (His)	0.19 ± 0.00	0.92 ± 0.04
Isoleucine (Ile)	0.21 ± 0.02	1.04 ± 0.06
Leucine (Leu)	0.26 ± 0.01	1.54 ± 0.09
Lysine (Lys)	0.59 ± 0.01	1.77 ± 0.10
Methionine (Met)	0.04 ± 0.03	0.57 ± 0.02
Phenylalanine (Phe)	0.29 ± 0.15	2.05 ± 0.09
Proline (Pro)	0.41 ± 0.02	2.32 ± 0.10
Serine (Ser)	1.32 ± 0.00	2.72 ± 0.14
Threonine (Thr)	0.19 ± 0.00	1.78 ± 0.09
Tryptophan (Trp)	0.25 ± 0.02	N.D.
Tyrosine (Tyr)	0.65 ± 0.22	1.48 ± 0.08
Valine (Val)	0.42 ± 0.01	1.79 ± 0.07
EAA (% total)	37.12 ± 0.46	33.70 ± 0.11
BCAA (Val + Leu + Ile) (% total)	10.18 ± 0.06	11.68 ± 0.05
AAA (Phe + Tyr + Trp) (% total)	13.60 ± 3.30	9.44 ± 0.39

EAA, essential amino acids; BCAA, branched-chain amino acids; AAA, aromatic amino acids. N.D., not determined. Results are expressed as mean ± SD (*n* = 2).

**Table 2:** Food, fiber (3.9 % of fiber from diet) and water intakes of control rats (*n* = 15) and rats treated with CSE (1 g/kg b.w.) (*n* = 15) during repeated dose toxicity study.

	Male		Female	
	Control	CSE	Control	CSE
*Food* (*g*/*rat*/*day*)				
Week 1	14.1 ± 2.8 ^a^	13.3 ± 1.4 ^a^	15.5 ± 3.4 ^a^	11.9 ± 0.9 ^a^
Week 2	17.5 ± 3.0 ^bc^	18.3 ± 1.2 ^c^	15.2 ± 1.1 ^ab^	14.2 ± 1.0 ^a^
Week 3	21.3 ± 1.2 ^b^	21.0 ± 2.7 ^b^	17.7 ± 4.4 ^ab^	16.2 ± 2.1 ^a^
Week 4	22.3 ± 0.9 ^b^	22.0 ± 1.0 ^b^	16.5 ± 1.7 ^a^	17.2 ± 1.7 ^a^
*Fiber intake* (*g*/*rat*/*day*)				
From diet	0.9 ± 0.0 ^a^	0.9 ± 0.0 ^a^	0.7 ± 0.1 ^a^	0.7 ± 0.1 ^a^
From CSE	-	0.1 ± 0.0	-	0.1 ± 0.0
Total	0.9 ± 0.0 ^a^	1.0 ± 0.0 ^b^	0.7 ± 0.1 ^a^	0.8 ± 0.1 ^a^
*Water (ml/rat/day)*				
Week 1	21.6 ± 5.3 ^a^	27.4 ± 9.2 ^a^	24.1 ± 4.2 ^a^	25.3 ± 9.0 ^a^
Week 2	33.1 ± 9.8 ^a^	30.1 ± 5.8 ^a^	26.6 ± 8.6 ^a^	31.9 ± 7.3 ^a^
Week 3	43.0 ± 7.5 ^a^	39.3 ± 9.9 ^a^	32.9 ± 4.7 ^a^	38.6 ± 5.7 ^a^
Week 4	37.9 ± 6.3 ^b^	41.0 ± 4.2 ^b^	28.0 ± 4.2 ^a^	35.5 ± 3.8 ^b^

Data are expressed as the means ± standard deviation. Values in each row with different letters differ significantly (Tukey test, *p* < 0.05).

**Table 3:** Body weights, absolute and relative organ weights (grams) of control rats (*n* = 15) and rats treated with CSE (1 g/kg) (*n* = 15).

	Male		Female	
Parameter	Control	CSE	Control	CSE
Body weight (g)	263.8 ± 15.0 ^b^	261.7 ± 18.5 ^b^	197.2 ± 10.4 ^a^	191.4 ± 12.8 ^a^
*Absolute organ weights* (*g*)				
Thymus	0.4 ± 0.1 ^a^	0.6 ± 0.2 ^a^	0.6 ± 0.2 ^a^	0.5 ± 0.1 ^a^
Lungs	1.7 ± 0.4 ^ab^	2.1 ± 0.4 ^b^	1.2 ± 0.6 ^a^	1.5 ± 0.3 ^ab^
Liver	10.0 ± 1.5 ^b^	9.2 ± 1.3 ^b^	6.6 ± 0.6 ^a^	7.2 ± 1.0 ^a^
Kidneys	1.0 ± 0.1 ^ab^	1.4 ± 0.6 ^b^	0.8 ± 0.1 ^a^	0.8 ± 0.1 ^a^
Adrenal glands	0.2 ± 0.0 ^a^	0.2 ± 0.1 ^a^	0.3 ± 0.1 ^a^	0.2 ± 0.1 ^a^
Sex organs	2.5 ± 0.2 ^b^	2.7 ± 0.4 ^b^	1.4 ± 0.3 ^a^	1.1 ± 0.3 ^a^
Brain	1.6 ± 0.1 ^a^	1.6 ± 0.2 ^a^	1.6 ± 0.2 ^a^	1.5 ± 0.3 ^a^
Heart	1.0 ± 0.1 ^b^	0.9 ± 0.1 ^ab^	0.8 ± 0.2 ^ab^	0.7 ± 0.1 ^a^
Spleen	0.5 ± 0.1 ^a^	0.4 ± 0.1 ^a^	0.5 ± 0.2 ^a^	0.5 ± 0.1 ^a^
*Relative organ weights* (*g*%)				
Thymus	0.2 ± 0.0 ^a^	0.2 ± 0.1 ^ab^	0.3 ± 0.1 ^b^	0.2 ± 0.0 ^ab^
Lungs	0.6 ± 0.2 ^a^	0.8 ± 0.1 ^a^	0.6 ± 0.3 ^a^	0.8 ± 0.1 ^a^
Liver	3.8 ± 0.4 ^a^	3.5 ± 0.4 ^a^	3.3 ± 0.3 ^a^	3.7 ± 0.4 ^a^
Kidneys	0.4 ± 0.0 ^a^	0.5 ± 0.2 ^a^	0.4 ± 0.0 ^a^	0.4 ± 0.1 ^a^
Adrenal glands	0.1 ± 0.0 ^a^	0.1 ± 0.0 ^a^	0.1 ± 0.1 ^a^	0.1 ± 0.1 ^a^
Sex organs	0.9 ± 0.1 ^b^	1.0 ± 0.2 ^b^	0.7 ± 0.2 ^a^	0.6 ± 0.2 ^a^
Brain	0.6 ± 0.0 ^a^	0.6 ± 0.1 ^a^	0.8 ± 0.1 ^b^	0.8 ± 0.1 ^b^
Heart	0.4 ± 0.0 ^a^	0.3 ± 0.0 ^a^	0.4 ± 0.1 ^a^	0.4 ± 0.1 ^a^
Spleen	0.2 ± 0.0 ^a^	0.2 ± 0.0 ^a^	0.3 ± 0.1 ^a^	0.2 ± 0.1 ^a^

Data are expressed as the means ± standard deviation. The values in each row with different letters differ significantly (Tukey test, *p* < 0.05).

**Table 4:** Clinical biochemistry and hematology of blood samples from control rats (*n* = 15) and rats treated with CSE (1 g/kg) (*n* = 15).

	**Male**		**Female**	
	**Control**	**CSE**	**Control**	**CSE**
*BIOCHEMICAL ANALYSES*				
Glucose (mg/dL)	109.7 ± 42.2 ^a^	101.0 ± 12.3 ^a^	117.7 ± 7.9 ^a^	106.0 ± 16.9 ^a^
Urea (mg/dL)	41.2 ± 3.5 ^ab^	38.5 ± 4.0 ^a^	46.1 ± 6.4 ^b^	39.2 ± 5.2 ^ab^
Creatinine (mg/dL)	<0.5 ^a^	<0.5 ^a^	<0.5 ^a^	<0.5 ^a^
Proteins (g/dL)	6.0 ± 0.7 ^a^	5.9 ± 0.4 ^a^	6.1 ± 0.3 ^a^	5.5 ± 0.2 ^a^
ALT (U/L)	32.4 ± 6.6 ^b^	26.6 ± 4.3 ^ab^	23.1 ± 5.4 ^a^	23.5 ± 3.5 ^a^
GGT (U/L)	<5.0 ^a^	<5.0 ^a^	<5.0 ^a^	<5.0 ^a^
Cholesterol (mg/dL)	104.8 ± 4.5 ^a^	103.3 ± 5.9 ^a^	102.3 ± 3.6 ^a^	105.6 ± 4.8 ^a^
Potassium (mEq/L)	3.5 ± 0.2 ^bc^	3.7 ± 0.5 ^c^	2.8 ± 0.1 ^a^	3.2 ± 0.3 ^ab^
Sodium /mEq/L)	144.0 ± 4.6 ^a^	145.2 ± 1.0 ^a^	144.0 ± 1.1 ^a^	142.2 ± 1.4 ^a^
Albumin (g/dL)	3.4 ± 0.1 ^ab^	3.3 ± 0.1 ^a^	3.5 ± 0.2 ^b^	3.2 ± 0.1 ^a^
Bile acid (μmol/mL)	20.7 ± 10.9 ^a^	15.0 ± 11.6 ^a^	11.7 ± 4.5 ^a^	14.0 ± 9.9 ^a^
*ERYTHROCYTE PARAMETERS*				
RBCs (×10^6^/µL)	8.5 ± 0.5 ^b^	8.1 ± 0.3 ^ab^	7.9 ± 0.4 ^ab^	7.8 ± 0.4 ^a^
Hemoglobin (g/dL)	16.2 ± 0.4 ^b^	16.1 ± 0.5 ^b^	15.2 ± 0.6 ^a^	14.8 ± 0.6 ^a^
HCT (%)	48.4 ± 1.5 ^b^	47.4 ± 1.2 ^b^	44.5 ± 2.5 ^a^	43.3 ± 2.3 ^a^
MCV (fl)	56.6 ± 2.4 ^ab^	58.3 ± 2.4 ^b^	56.2 ± 0.9 ^ab^	55.3 ± 1.4 ^a^
MCH (pg)	19.0 ± 0.8 ^a^	19.8 ± 0.8 ^a^	19.2 ± 0.3 ^a^	19.0 ± 0.8 ^a^
MCHC (g/dL)	33.6 ± 0.3 ^a^	34.1 ± 0.7 ^a^	34.2 ± 0.6 ^a^	34.3 ± 0.8 ^a^
RDW (%)	11.8 ± 0.6 ^b^	11.5 ± 0.4 ^ab^	11.1 ± 0.3 ^a^	11.6 ± 0.4 ^ab^
*LEUKOCYTE PARAMETERS*				
WBCs (×10^3^/µL)	8.1 ± 1.4 ^b^	7.1 ± 3.0 ^ab^	4.7 ± 1.4 ^a^	4.6 ± 1.4 ^a^
Segmented Neutrophils (μL)	1155.0 ± 303.5 ^b^	586.3 ± 304.4 ^a^	864.4 ± 396.2 ^ab^	499.0 ± 226.2 ^a^
Band Neutrophils (μL)	N.D.	N.D.	N.D.	N.D.
Lymphocytes (µL)	6919.0 ± 1323.6 ^c^	6395.7 ± 2738.7 ^bc^	3683.0 ± 1132.9 ^a^	3996.8 ± 1550.2 ^ab^
Monocytes (µL)	37.7 ± 47.5 ^a^	71.0 ± 67.0 ^a^	82.8 ± 82.3 ^a^	57.1 ± 42.2 ^a^
Eosinophils (μL)	59.7 ± 66.5 ^a^	84.3 ± 98.7 ^a^	69.2 ± 94.9 ^a^	75.5 ± 18.4 ^a^
Basophils (μL)	N.D.	N.D.	43.2 ± 93.9	N.D.
*PLATELET PARAMETERS*				
Platelets (×10^3^ /µL)	801.8 ± 213.9 ^a^	773.5 ± 309.3 ^a^	763.0 ± 272.9 ^a^	755.4 ± 368.4 ^a^
MPV (fl)	9.0 ± 0.5 ^a^	9.0 ± 0.8 ^a^	8.7 ± 0.6 ^a^	9.1 ± 0.4 ^a^
Plateletcrit (%)	0.7 ± 0.1 ^a^	0.6 ± 0.2 ^a^	0.6 ± 0.2 ^a^	0.6 ± 0.3 ^a^

N.D. Non detected. Data are expressed as the means ± standard deviation. The values in each row with different letters differ significantly (Tukey test, *p <* 0.05). ALT, alanine aminotransferase; CSE, coffee silverskin extract; GGT, gamma-glutamyl transferase; HCT, hematocrit; MCH, mean corpuscular hemoglobin; MCHC, mean corpuscular hemoglobin concentration; MCV, mean corpuscular volume; MPV, mean platelet volume; RBCs, red blood cells; RDW, red cell distribution width; WBCs, white blood cells.

**Table 5:** Analyses of serum from control rats (*n* = 15) and rats treated with CSE (1 g/kg) (*n* = 15).

	Male		Female	
	Control	CSE	Control	CSE
*Oxidative stress*				
Total antioxidant capacity (Eq. Trolox mM)	2.0 ± 0.1 ^a^	2.0 ± 0.1 ^a^	2.0 ± 0.0 ^a^	2.0 ± 0.1 ^a^
Glutathione Peroxidase (GPx) (U/mL)	0.2 ± 0.0 ^a^	0.2 ± 0.0 ^a^	0.2 ± 0.0 ^a^	0.2 ± 0.0 ^a^
Glutathione Reductase (GR) (U/mL)	3.5 ± 0.8 ^a^	3.9 ± 1.2 ^a^	4.1 ± 0.9 ^a^	3.7 ± 0.9 ^a^
Superoxide Dismutase (SOD) (U/mL)	2.9 ± 0.8 ^a^	3.1 ± 1.4 ^a^	3.0 ± 1.3 ^a^	3.2 ± 1.3 ^a^
Catalase (CAT) (U/mL)	7.0 ± 4.2 ^a^	7.4 ± 5.1 ^a^	5.3 ± 1.5 ^a^	7.1 ± 4.3 ^a^
*Inflammation*				
C Reactive Protein (μL U/mL)	0.1 ± 0.0 ^a^	0.1 ± 0.0 ^a^	0.1 ± 0.0 ^a^	0.1 ± 0.0 ^a^
*Hormones*				
Insulin (ng/mL)	34.9 ± 19.3 ^a^	29.4 ± 28.8 ^a^	21.9 ± 12.6 ^a^	26.9 ± 8.9 ^a^
Serotonin (ng/mL)	240.8 ± 43.8 ^a^	239.1 ± 46.0 ^a^	208.4 ± 26.4 ^a^	210.7 ± 20.2 ^a^
Melatonin (pg/mL)	671.9 ± 258.1 ^a^	853.4 ± 161.5 ^a^	784.5 ± 196.3 ^a^	900.6 ± 45.4 ^a^

Data are expressed as the means ± standard deviation. Values in each row with different letters differ significantly (Tukey test, *p* < 0.05).

**Table 6:** Analyses of feces generated during 24 h from control rats (*n* = 15) and rats treated with CSE (1 g/kg) (*n* = 15) collected on the last day of the study.

	Male		Female	
	Control	CSE	Control	CSE
Number pellet/rat	53.0 ± 0.5 ^b^	44.0 ± 1.6 ^a^	44.0 ± 0.1 ^a^	41.0 ± 2.0 ^a^
Feces weight (g)/pellet	0.04 ± 0.0 ^a^	0.03 ± 0.0 ^a^	0.03 ± 0.0 ^a^	0.03 ± 0.0 ^a^
Feces weight (g)/rat	5.5 ± 0.2 ^b^	5.2 ± 0.0 ^ab^	4.2 ± 0.4 ^a^	4.4 ± 0.2 ^a^
pH	6.3 ± 0.1 ^a^	6.3 ± 0.1 ^a^	6.3 ± 0.1 ^a^	6.3 ± 0.1 ^a^
Total antioxidant capacity (Eq. Trolox mM)	8.2 ± 0.4 ^a^	7.8 ± 0.3 ^a^	8.1 ± 0.4 ^a^	7.8 ± 0.7 ^a^
**SCFAs (µmol/g)**				
Acetic	46.42 ± 1.75 ^a^	68.49 ± 8.23 ^c^	59.64 ± 1.79 ^b^	62.18 ± 4.22 ^bc^
Propionic	5.14 ± 0.24 ^a^	5.94 ± 0.90 ^a^	7.09 ± 0.34 ^b^	5.29 ± 0.38 ^a^
Isobutyric	0.24 ± 0.01 ^a^	0.28 ± 0.04 ^ab^	0.40 ± 0.02 ^c^	0.31 ± 0.03 ^b^
Butyric	6.71 ± 1.08 ^c^	2.33 ± 0.20 ^a^	10.63 ± 0.73 ^d^	4.51 ± 0.34 ^b^
Isovaleric	0.17 ± 0.02 ^a^	0.16 ± 0.03 ^a^	0.28 ± 0.01 ^c^	0.21 ± 0.02 ^b^
Valeric	0.49 ± 0.05 ^b^	0.40 ± 0.06 ^a^	0.78 ± 0.03 ^c^	0.48 ± 0.04 ^b^
Caproic	0.40 ± 0.04 ^b^	0.30 ± 0.04 ^a^	0.92 ± 0.03 ^d^	0.55 ± 0.06 ^c^
Heptanoic	N.D.	N.D.	N.D.	N.D.
Total SCFAs	59.57 ± 3.14 ^a^	77.90 ± 9.42 ^b^	79.75 ± 2.81 ^b^	73.52 ± 5.05 ^b^

Data are expressed as the means ± standard deviation. Values in each row with different letters differ significantly (Tukey test, *p* < 0.05).

## Supplementary Materials

The following are available online at https://www.mdpi.com/2072-6643/11/11/2693/s1, Table S1: Fat (%) and fatty acid content (g/100 g of FA methyl esters) of coffee silverskin extract (CSE).
