# Supplementary material for: Coffee Silverskin Extract: Nutritional Value, Safety and Effect on Key Biological Functions
Source: Nutrients. 2019 Nov 7;11(11):2693. doi: 10.3390/nu11112693 (PMC6893552; doi:10.3390/nu11112693)
Supplement: Supplementary file 1 [file nutrients-11-02693-s001.pdf]

nutrients MDPI

Table S1. Fat (%) and fatty acid content (g/100 g of FA methyl esters) of coffee silverskin extract (CSE).

	CSE
**Fat (%)**	0.44 ± 0.14
**Fatty acid profile (g/100 g)**	
C14:0	1.24 ± 0.03
C15:0	0.52 ± 0.00
C16:0	26.24 ± 0.02
C16:1n7	0.26 ± 0.01
C17:0	0.16 ± 0.01
C18:0	6.54 ± 0.02
C18:1n7c	0.84 ± 0.01
C18:1n9c	5.44 ± 0.10
C18:2n6c	20.89 ± 0.11
C18:3n3	0.96 ± 0.04
C20:0	12.86 ± 0.04
C20:1n9	0.37 ± 0.00
C20:5n3	0.18 ± 0.01
C21:0 / C20:3n6*	0.32 ± 0.00
C22:0	18.74 ± 0.04
C22:6n3	0.29 ± 0.07
C23:0	0.50 ± 0.01
C24:0 /C22:5n3*	3.63 ± 0.03
SFA	66.81 ± 0.01
MUFA	6.92 ± 0.10
PUFA	25.95 ± 0.11

SFA, saturated fatty acids; MUFA, monounsaturated fatty acids; PUFA, polyunsaturated fatty acids. Results are expressed as mean ± SD (n = 3).
